# Limitations of Using IL-17A and IFN-γ-Induced Protein 10 to Detect Bovine Tuberculosis

**DOI:** 10.3389/fvets.2018.00028

**Published:** 2018-03-06

**Authors:** Ting Xin, Xintao Gao, Hongjun Yang, Pingjun Li, Qianqian Liang, Shaohua Hou, Xiukun Sui, Xiaoyu Guo, Weifeng Yuan, Hongfei Zhu, Jiabo Ding, Hong Jia

**Affiliations:** ^1^Institute of Animal Sciences (IAS), Chinese Academy of Agricultural Sciences (CAAS), Beijing, China; ^2^China Institute of Veterinary Drugs Control, Beijing, China; ^3^Dairy Cattle Research Center, Shandong Academy of Agricultural Sciences, Jinan, China; ^4^Molecular and Cellular Biology, Gembloux Agro-Bio Tech University of Liège (ULg), Gembloux, Belgium

**Keywords:** bovine tuberculosis, IFN-γ-induced protein 10, IL-17A, interferon gamma, nested PCR

## Abstract

Bovine tuberculosis (bTB) is primarily caused by infection with *Mycobacterium bovis*, which belongs to the *Mycobacterium tuberculosis* complex. The airborne route is considered the most common for transmission of *M. bovis*, and more than 15% of cattle with bTB shed the *Mycobacterium*, which can be detect by nested PCR to amplify mycobacterial *mpb*70 from a nasal swab from a cow. To screen for cytokines fostering early and accurate detection of bTB, peripheral blood mononuclear cells were isolated from naturally *M. bovis*-infected, experimentally *M. bovis* 68002-infected, and uninfected cattle, then these cells were stimulated by PPD-B, CFP-10-ESAT-6 (CE), or phosphate-buffered saline (PBS) for 6 h. The levels of interferon gamma (IFN-γ), IFN-γ-induced protein 10 (IP-10), IL-6, IL-12, IL-17A, and tumor necrosis factor alpha mRNA were measured using real-time PCR. To explore the cytokines associated with different periods of *M. bovis* infection, cattle were divided into three groups: PCR-positive, PCR-negative, and uninfected using the tuberculin skin test, CFP-10/ESAT-6/TB10.4 protein cocktail-based skin test, IFN-γ release assay (IGRA), CFP-10/ESAT-6 (CE)-based IGRA, and nested PCR. The expression of IP-10, IL-17A, and IFN-γ proteins induced by PPD-B, CE, or PBS was detected by ELISA. The results showed that levels of PPD-B-stimulated IL-17A and IP-10 (mRNA and protein), and CE-induced IP-10 (mRNA and protein) were significantly higher in cattle naturally or experimentally infected with *M. bovis* than in those that were uninfected. The levels of PPD-B- or CE-induced IL-17A and IP-10 (protein) could be used to differentiate *M. bovis*-infected calves from uninfected ones for 6 to 30 weeks post-infection, whereas PPD-B- and CE-induced IP-10 and IL-17A mRNA expression could be used to differentiate *M. bovis*-infected calves from uninfected ones between 6 and 58 weeks post-infection. However, CE-induced IL-17A (protein) was not a reliable indicator of *M. bovis* infection in cattle that were confirmed positive for infection by nested PCR. Furthermore, the levels of PPD-B- or CE-induced IP-10 and IL-17A protein were lower than IFN-γ in *M. bovis*-infected cattle. Therefore, IL-17A and IP-10 protein are not suitable biomarkers for bTB. Antigen-induced IP-10 mRNA should be analyzed further for their potential to be used in the diagnosis of bTB.

## Introduction

Bovine tuberculosis (bTB) is primarily caused by infection with *Mycobacterium bovis*, which belongs to the *Mycobacterium tuberculosis* complex (MTC) that includes *M. tuberculosis, M. bovis, M. africanum, M. microti, M. caprae, Mycobacterium pinnipedii, M. mungii*, and *M. canetti* (1, 2). *M. bovis* could infect wild animals and has a relatively wider host range than *M. tuberculosis* (3). Humans can also be infected with *M. bovis* through the ingestion of raw milk products or inhalation of aerosols (4). The airborne route of infection is considered the most common for transmission of *M. bovis*, and more than 15% of cattle infected with *M. bovis* shed the *Mycobacterium* (5), mainly early in infection (6). Therefore, early and accurate detection of bTB is important to control transmission of *M. bovis* spreading to other animals (7).

The traditional bTB diagnostic methods are the tuberculin skin test (TST) and interferon gamma (IFN-γ) release assay (IGRA). Both TST and IGRA are based on the detection and comparison of cell-mediated responses induced by bovine purified protein derivatives (PPD-B) and avian purified protein derivatives (PPD-A) (8). PPD-B is obtained from a culture of virulent *M. bovis*, but shared antigens with non-pathogenic environmental *mycobacteria* can reduce the specificity of the TST (9). Although PPD-A is used in IGRA to exclude environmental *Mycobacterium* infections, it has failed to detect some *M. bovis*-infected cattle in bTB- and paratuberculosis- coprevalent dairies. Therefore, TST and IGRA based on *M. bovis-*specific antigens such as CFP-10 and ESAT-6 were established to obtain higher specificity in the diagnosis of bTB (8–11). Considering that neither TST nor IGRA can differentiate between stages in the progression of bTB, a nested PCR assay based on the amplification of a fragment of *mpb*70 was established and used to detect mycobacteria in milk, nasal exudates, and bronchoalveolar lavage (BAL) fluid (12).

To develop more effective and accurate methods to diagnose tuberculosis, new candidate biomarkers have emerged recently for the diagnosis of this disease in humans and cattle. These potential biomarkers include tumor necrosis factor alpha (TNF-α), interleukin-2, IL-1β, IL-17A, and IFN-γ-induced protein 10 (IP-10). IP-10 and IL-17A, particularly, have the potential to be used to differentiate between active and latent TB in humans. However, there are only a few studies on screening for bTB-related cytokines.

In this study, we screened for cytokines related to bTB and explored whether cytokines could be used to detect *M. bovis*-infected cattle [including those that were PCR-positive (PCR-P) and those that were PCR-negative (PCR-N)]. First, we isolated peripheral blood mononuclear cells (PBMCs) from naturally *M. bovis*-infected, from experimentally *M. bovis* 68002-infected and from uninfected cattle, and then we stimulated the PBMCs using PPD-B, CFP-10-ESAT-6 (CE), or phosphate-buffered saline (PBS) for 6 h. The levels of IFN-γ, IP-10, IL-6, IL-12, IL-17A, and TNF-α mRNA transcripts were determined using real-time PCR. To explore whether IP-10 and IL-17A can be used to detect all *M. bovis-*infected cattle, cattle were divided into three groups: nested PCR-P, nested PCR-N, and uninfected cattle as determined by TST, CFP-10/ESAT-6/TB10.4 protein cocktail-based skin test, IGRA, CE-based IGRA, and nested PCR. The expression of IP-10, IL-17A, and IFN-γ proteins induced by PPD-B or CE was detected by ELISA.

## Materials and Methods

### Ethics Approval

The six Luxi beef calves used in the present study were treated carefully and according to the protocol approved by the Animal Care and Use Committee of the China Institute of Veterinary Drug Control. The animal ethics committee approval number is SYXK (2005-0021).

### Bacterial Species and Plasmids

*Mycobacterium bovis* 68002 was isolated in China and preserved in the China Institute of Veterinary Drug Control, Beijing, China. *M. bovis* 68002 can cause tubercles in the lungs, liver and other organs after intravenous injection into calves. It was defined as highly virulent and used to prepare bovine tuberculin in China. Bovine tuberculin (PPD-B, Harbin Pharmaceutical Group, Heilongjiang Province, China) was used in the TST. The CFP-10/ESAT-6/TB10.4 protein cocktail (0.5 mg/ml, endotoxin less than 10 EU/mg) was prepared in the Institute of Animal Sciences (IAS-CAAS), and used to make the protein cocktail-based skin test. CFP-10-ESAT-6 (20 μg/ml, CE expressed and purified in our lab, with a Trx-His-S tag at the N-terminus, with endotoxin at a concentration less than 10 EU/mg) was used in the CE-based IGRA. PET [20 μg/ml, the tag protein of the pET(32a) + vector was expressed and purified in our lab, with endotoxin at a concentration of less than 10 EU/mg] was used as control for CE. Bovine IFN-γ (500 μg/ml, expressed and purified in our lab, with a 6-His tag at the N-terminus, with endotoxin at a concentration less than 10 EU/mg) was used as a reference standard in the IGRA for the quantitative analysis of IFN-γ in bovine plasma.

### Skin Test Procedure

The TST was performed as the Chinese diagnostic standard for bTB (GB/T 18645-2002), and the CFP-10/ESAT-6/TB10.4 protein cocktail-based skin test was previously established in our laboratory. As described in an earlier report (9), PPD-B (PPD-B, 2,500 IU/cattle) and the CFP-10/ESAT-6/TB10.4 protein cocktail were intradermally injected (0.1 ml each) into two sites on the same side of a cow’s neck. Differences in skin thicknesses (mm) pre- and 72 h post-injection were calculated. With the GB/T 18645-2002, if the difference in skin thicknesses was ≥4 mm, the cattle were considered as *M. bovis*-infected; if the difference in skin thicknesses was <2 mm, the cattle were considered *M. bovis*-uninfected; if the difference in skin thicknesses then were between 2 and 4 mm, the cattle were suspected to be *M. bovis*-infected, and were retested after an interval of 60 days. If the difference in skin thicknesses was ≥2 mm, the cattle were considered *M. bovis*-infected. The cutoff value for the CFP-10/ESAT-6/TB10.4 protein cocktail-based skin test was obtained by ROC analysis based on 125 uninfected cattle and 117 *M. bovis*-infected cattle, whose results were confirmed by nested PCR (9). For the CFP-10/ESAT-6/TB10.4 protein cocktail-based skin test, if the difference in skin thicknesses was ≥1.1 mm, the cattle were considered *M. bovis-*infected; if the difference in skin thicknesses was <1.1 mm, the cattle were considered free from bTB.

### Bovine IFN-γ, IP-10, and IL-17A ELISAs

All cattle tested by skin test were also tested by IGRA (*Mycobacterium bovis* Gamma Interferon Test Kit for cattle, Bovigam, Prionics AG, Schlieren, Switzerland) according to the manufacturer’s instructions. Briefly, heparinized blood was collected from each cow and dispensed into a 24-well cell culture plate (1.5 ml/well, five wells for each cow), and stimulated with 100 µl of PPD-A (Avian Tuberculin PPD, 300 µg/ml, Prionics AG, Schlieren, Switzerland), PPD-B (Bovine Tuberculin PPD, 300 µg/ml, Prionics AG, Schlieren, Switzerland), PBS, PET, and CE, respectively. Plasma was collected from each well after incubation for 24 h at 37°C in 5% CO_2_.

The IFN-γ in plasma was detected using a *Mycobacterium bovis* Gamma Interferon Test Kit. PPD-B-simulated blood plasma having an OD value more than 0.100 above that of plasma stimulated with PPD-A and PBS, and CE-stimulated blood plasma having an OD value more than 0.100 above that of plasma stimulated with PBS indicated cattle were *M. bovis*-infected. The cutoff value for the CE-based IGRA was obtained in our lab by ROC analysis based on 96 uninfected cattle and 258 *M. bovis*-infected cattle, whose results were confirmed by nested PCR. The IFN-γ in plasma could be quantified using recombinant bovine IFN-γ as the reference standard.

IFN-γ-induced protein 10 and IL-17A in plasma were detected using Bovine IP-10 ELISA VetSet (Kingfisher Biotech Inc., Saint Paul, MN, USA) and Bovine IL-17A ELISA VetSet (Kingfisher Biotech Inc., Saint Paul, MN, USA).

### Nested PCR Analysis

Nested PCR was conducted to confirm the *M. bovis* infection as reported before (11). Briefly, nasal swabs of each cow were collected, and immersed immediately into 2 ml of sterile PBS. Then, DNA was extracted and dissolved in 20 µl of Tris buffer, and a nested PCR was performed to amplify a region of the *mpb70* gene of MTC bacteria as described previously.

### Analysis of Cytokine Gene Expression by Real-time PCR

Peripheral blood mononuclear cells were isolated from heparinized blood using the density gradient centrifugation method with Ficoll-Hypaque Density Solution (TIAN JIN HAO YANG Biological Manufacture Co., Ltd., Tianjin). Red blood cells (RBCs) were separated from PBMCs by RBC lysis buffer (Invitrogen, USA). After that, PBMCs were washed with PBS three times and resuspended in RPMI1640 medium [with 10% (vol/vol) fetal bovine serum, 25 mM HEPES buffer, 2 mM l-glutamine, 100 U/ml of penicillin, and 0.1 mg/ml streptomycin]. The PBMCs were seeded into a 48-well cell culture plate at 4 × 10^6^ cells in a total volume of 250 µl. Following isolation, PBMCs were subsequently incubated at 37°C in 5% CO_2_ with PPD-B, CE, PET, or PBS for 6–8 h. After incubation, the cells were immediately lysed with 750 µl of TRIzol (Invitrogen, USA), and total RNA was isolated using an RNAeasy Mini Kit (Qiagen, Valencia, CA, USA) according to the manufacturer’s protocol and eluted from the column with 50 µl of DEPC water. One microliter of RNA inhibitor (RI) was added to the RNA solution to protect against RNAase. The RNA was quantified with a spectrophotometer. One microgram of RNA was pre-mixed with 0.5 µg of Oligo dT (15) (Promega, USA) and DEPC water. A total of 12 µl of the pre-mixture was warmed in a water bath at 70°C for 5 min, and then immediately cooled in an ice bath for 5 min. The full-length cDNA was reversely transcribed in a 25-µl reaction mixture containing a pre-mixture of 5 µl of 5 × MLV Buffer (TaKaRa, Japan), 1.25 µl of 10 mM dNTP (TaKaRa, Japan), 0.5 µl of RI (TaKaRa, JPN), 1 µl of M-MLV (Promega, USA), and 5.25 µl of DEPC water. The mixture was incubated at 30°C for 10 min, followed by 42°C for 60 min, and then at 70°C for 5 min. The cDNA was stored at −80°C.

Real-time PCR was carried out with TaqMan^®^ Real-Time PCR Master Mix (ABI). The primer and probe sequences (Table 1) were designed and synthesized by Shanghai GengCore Bio-technologies Co., Ltd. All reactions were performed in a 25-µl volume containing 10 µl of TaqMan Real-Time PCR Master Mix, 0.2 µl of Primer F (10 µM), 0.2 µl of Primer R (10 µM), 0.2 µl of Taqman-Probe (20 µM), and 1 µl of template cDNA. All reactions were run in triplicate and were carried out in 96-well Rxn Plate (ABI, USA) sealed with optical adhesive film (ABI, USA) on an ABI7900HT Fast Real-Time PCR System (ABI, USA). The instrument was programmed to cycle at 95°C for 2 min, followed by 40 cycles of 15 s at 95°C and then 1 min at 60°C. Relative gene expression was calculated using the 2^−ΔΔ^*^CT^* method, with *β-actin* as the reference gene, and the PBS-stimulated sample from each cow was used to calibrate PBMCs responses.

**Table 1:** Primer and probe sequences used in real-time PCR.

Primer and probe	Sequences
β-actin-F	5′-GCCCTGAGGCTCTCTTCCA-3′
β-actin-R	5′-GCGGATGTCGACGTCACA-3′
β-actin-P	5′-FCATGGAATCCTGCGGCATTCACG-BHQ1-3′
IFN-γ-F	5′-GCTGATTCAAATTCCGGTGGA-3′
IFN-γ-R	5′-CAGGCAGGAGGACCATTACG-3′
IFN-γ-P	5′-FTCTGCAGATCCAGCGCAAAGCC-BHQ1-3′
IP-10-F	5′-GTCCTTAGAAAAACTTGAAGTCATTCC-3′
IP-10-R	5′-TTCTTGATGGTCTTAGATTCTGGATTC-3′
IP-10-P	5′-FCCCACGTGTCGAGATTATTGCCACAA-BHQ1-3′
IL-6-F	5′-AAATGGAGGAAAAGGACGGA-3′
IL-6-R	5′-TGATTTCCCTCATACTCGTTCTG-3′
IL-6-P	5′-FCTTCCAATCTGGGTTCAATCAGGCGA-BHQ1-3′
IL-12p40-F	5′-TGCACAAGCTCAAGTATGAAAACTA-3′
IL-12p40-R	5′-ACCTCCACCTGCCGAGAAT-3′
IL-12p40-P	5′-FCAGGGACATCATCAAACCAGACCCAC-BHQ1-3′
IL-17A-F	5′-AGAAGGCCCACCGATTATCA-3′
IL-17A-R	5′-CCACCTTCCCTTCAGCATTGA-3′
IL-17A-P	5′-FACTCTCCACCGCAATGAGGACCCTG-BHQ1-3′
TNF-α-F	5′-AGAAATTAGGGATGTAGGGAAGTGA-3′
TNF-α-R	5′-CTTGTGGACCCCAGGGAGTT-3′
TNF-α-P	5′-FTGGACAACGGGCCACCAACCA-BHQ1-3′

### Animals and Infection

To preliminarily screen for the cytokines related to *M. bovis* infection, 10 naturally *M. bovis*-infected and 5 uninfected Holstein cows were determined by TST, CFP-10/ESAT-6/TB10.4 protein cocktail-based skin test, IGRA, and CE-based IGRA. The PBMCs from each cow were isolated and treated with PPD-B, CFP-10-ESAT-6 (CE), or PBS for 6–8 h. The levels of IFN-γ, IP-10, IL-6, IL-12, IL-17A, and TNF-α mRNA transcripts were measured using real-time PCR.

To verify the cytokines related to *M. bovis* infection, six male Luxi beef calves aged 1–2 months from a bTB-free dairy farm were randomly selected for testing by TST, CFP-10/ESAT-6/TB10.4 protein cocktail-based skin test, IGRA, and CE-based IGRA, and maintained in a biosafety level-3 facility. Three calves were intravenously injected with 10^6^ C *M. bovis* 68002; another three calves were intravenously injected with PBS to serve as uninfected controls. Animals in each group were monitored for 1 year, and tested using TST and CFP-10/ESAT-6/TB10.4 protein cocktail-based skin test before injection, at 8 and 32 weeks post-infection. These six calves were also tested using IGRA and CE-based IGRA before injection, and at 8, 24, 48, 72, and 96 h, and 2, 4, 6, 8, 12, 16, 20, 26, 30, 34, 38, 45, 50, and 58 weeks post-infection. The levels of IFN-γ, IP-10, and IL-17A mRNA transcripts and protein induced by PPD-B, CE, PET, or PBS were determined before injection and at 6, 26, 30, and 58 weeks post-infection.

To explore whether PPD-B- or CE-induced IP-10, IP-17A, and IFN-γ proteins could be used to determine the stage of *M. bovis* infection, more than 1,000 Holstein cows were detected by TST, CFP-10/ESAT-6/TB10.4 protein cocktail-based skin test, IGRA, CE-based IGRA. The nasal swabs from *M. bovis*-infected cows were collected and detected by nested PCR, then the PCR-P swabs were confirmed by *mycobacteria* culture. The *M. bovis*-infected cows could be divided into two groups (Table 2): PCR-P (21 cows determined positive for *M. bovis* by TST, CFP-10/ESAT-6/TB10.4 protein cocktail-based skin test, IGRA, CE-based IGRA, and nested PCR were selected for the study) and PCR-N (21 cows determined positive for *M. bovis* by TST, CFP-10/ESAT-6/TB10.4 protein cocktail-based skin test, IGRA and CE-based IGRA, but negative by nested PCR were selected for the study). Heparinized whole blood from each cow was collected and stimulated with PPD-B, CE, or PBS for 24 h, and the plasma was collected from each well. The levels of IP-10, IL-17A, and IFN-γ protein expression in plasma were determined by ELISA.

**Table 2:** Characteristics of PCR-positive (PCR-P), PCR-negative (PCR-N) and NC groups.

Diagnostic methods	*Mycobacterium bovis*-infected cattle		NC
	PCR-P	PCR-N	
TST	+	+	−
CFP-10/ESAT-6/TB10.4 protein cocktail-based skin test	+	+	−
IGRA	+	+	−
CE-based IGRA	+	+	−
Nested PCR	+	−	−

*TST, tuberculin skin test; IGRA, interferon gamma (IFN-γ) release assay; CE-based IGRA, CFP-10/ESAT-6 (CE)-based IFN-γ release assay; PCR-P, cattle determined to be infected with M. bovis by nested PCR; PCR-N, cattle determined not to be infected with M. bovis by nested PCR; NC, cattle from a bovine tuberculosis-free dairy farm considered to be uninfected with M. bovis*.

The uninfected animals were from a bTB-free dairy farm and determined free from bTB by TST, CFP-10/ESAT-6/TB10.4 protein cocktail-based skin test, IGRA, CE-based IGRA, and nested PCR. All animals used in this study were determined free of paratuberculosis by *Mycobacterium paratuberculosis* Antibody Test Kit for Cattle (IDEXX Montpellier SAS, France) and free of brucellosis by Svanova Brucella-Ab C-ELISA (Svanova Biotech AB, Uppsala, Sweden).

### Bacteriological Analysis

Three *M. bovis* 68002-infected calves and one naturally *M. bovis*-infected cow were slaughtered after the experiment. Tissue samples from the lung, liver, spleen, bronchial lymph nodes, and kidney were collected and analyzed using nested PCR, *Mycobacterium* culture, and hematoxylin–eosin staining.

### Statistical Analysis

Data were analyzed by analysis of variance (ANOVA) followed by Kruskal–Wallis test or Spearman correlation using GraphPad Prism 5 software (San Diego, CA, USA). A *p* value of <0.05 (two-tailed) was considered significant.

## Results

### Screening of Cytokines Related to *M. bovis* Infection in Naturally *M. bovis*-Infected Cattle

**Figure 1:**
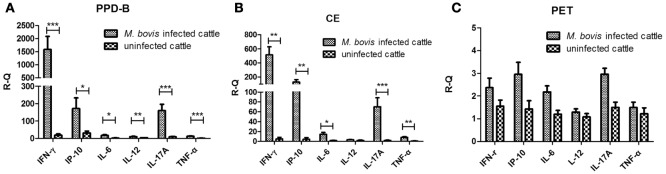
Levels of cytokine mRNA in *Mycobacterium bovis*-infected cattle and bovine tuberculosis (bTB)-free cattle. **(A)** Peripheral blood mononuclear cells (PBMCs) stimulated by PPD-B for 6 h. **(B)** PBMCs stimulated by CE for 6 h. **(C)** PBMCs stimulated by PET for 6 h. PBMCs were isolated from 10 cows naturally infected with *M. bovis* and 5 cows free of bTB, and these cells stimulated with PPD-B, CE, PET, or phosphate-buffered saline (PBS). RNA was isolated to measure the levels of interferon gamma (IFN-γ), IFN-γ-induced protein 10 (IP-10), IL-6, IL-12, IL-17A, and tumor necrosis factor alpha (TNF-α) gene expression using the 2^−ΔΔ^*^CT^* method, with PBS-stimulated PBMCs as a calibrator and *β-actin* as the reference gene. The significance between cytokines transcript levels in *M. bovis*-infected and bTB-free cattle was determined using an unpaired t test. *p < 0.05; **p < 0.001; ***p < 0.0001. Data are mean ± SEM values (for *M. bovis*-infected cattle, *n* = 10; for bTB-free cattle, *n* = 5).

To screen for cytokines related to *M. bovis* infection, 10 naturally *M. bovis*-infected and five uninfected cows were determined by TST, CFP-10/ESAT-6/TB10.4 protein cocktail-based skin test, IGRA, and CE-based IGRA. Levels of IFN-γ, IP-10, IL-6, IL-12, IL-17A, and TNF-α mRNA in PBMCs from each cow stimulated with PPD-B, CE, PET, or PBS were measured using real-time PCR. The levels of IFN-γ, IP-10, IL-6, IL-17A, and TNF-α mRNA were significantly higher in both PPD-B- and CE-stimulated PBMCs from *M. bovis-*infected cows than from uninfected cows (Figures 1A,B), whereas the levels of IL-12 mRNA were significantly higher only in PPD-B-stimulated PBMCs from *M. bovis*-infected cows relative to those in uninfected cows (Figure 1A). The transcript levels of these six cytokines induced by PET were similar to those exposed to PBS, and there was no difference in these levels between *M. bovis-*infected and -uninfected cows (Figure 1C). The transcript levels of these six cytokines induced by PPD-B in PBMCs from both *M. bovis-*infected and -uninfected cows were higher than those induced by CE (Figures 1A,B). The levels of IFN-γ, IP-10, and IL-17A transcripts induced by PPD-B or CE in PBMCs from *M. bovis-*infected cows were higher than those of IL-6, IL-12, and TNF-α.

Correlations among the levels of transcripts of the six cytokines were determined, and IFN-γ, IP-10, IL-17A, and TNF-α induced by PPD-B or CE showed good correlation with each other (Table 3). IL-6 mRNA induced by PPD-B or CE showed good correlation with IL-17A, IFN-γ, and TNF-α mRNA. The levels of PPD-B-induced IL-12 significantly correlated with those of IFN-γ, IP-10, IL-17A, and TNF-α, whereas the levels of CE-induced IL-12 only correlated with those of IL-6. TNF-α levels showed better correlation with IFN-γ (*r* = 0.88) and IL-17A (*r* = 0.74) levels than that with those of IL-6 (*r* = 0.66) or IP-10 (*r* = 0.61).

**Table 3:** Correlations between interferon gamma (IFN-γ), IFN-γ-induced protein 10 (IP-10), IL-6, IL-12, IL-17A, and tumor necrosis factor alpha (TNF-α) (mRNA) induced by PPD-B and CE.

Correlation coefficient (Spearman *r*)	PPD-B-stimulated peripheral blood mononuclear cells (PBMCs)						CE-stimulated PBMCs					
	IFN-γ	IP-10	IL-6	IL-12	IL-17A	TNF-α	IFN-γ	IP-10	IL-6	IL-12	IL-17A	TNF-α
IFN-γ		0.75*	0.53*	0.70*	0.73*	0.80*		0.65*	0.66*	0.47	0.79*	0.88*
IP-10	0.75*		0.41	0.71*	0.56*	0.65*	0.65*		0.44	0.15	0.79*	0.61*
IL-6	0.53*	0.41		0.46	0.83*	0.62*	0.66*	0.44		0.58*	0.83*	0.66*
IL-12	0.70*	0.71*	0.46		0.55*	0.84*	0.47	0.15	0.58*		0.50	0.45
IL-17A	0.73*	0.56*	0.83*	0.55*		0.79*	0.79*	0.79*	0.83*	0.50		0.74*
TNF-α	0.80*	0.65*	0.62*	0.84*	0.79*		0.88*	0.61*	0.66*	0.45	0.74*	

PBMCs were isolated from naturally *Mycobacterium bovis* infected and bovine tuberculosis-free cattle, and stimulated with PPD-B, CE, PET, or phosphate-buffered saline (PBS). RNA was isolated to measures levels IFN-γ, IP-10, IL-6, IL-12, IL-17A, and TNF-α gene expression using the 2^−ΔΔ^*^CT^* method, with PBS-stimulated PBMCs used as a calibrator, and *β-actin* as the reference gene. Correlation coefficients were determined using Spearman’s two-tailed correlation test. *p < 0.05, significant correlation. Bovine Tuberculin PPD, 300 µg/ml (Prionics AG, Schlieren, Switzerland). CE: CFP-10-ESAT-6, 20 μg/ml, expressed and purified in our lab, with a Trx-His-S tag at the N-terminus, with endotoxin at a concentration less than 10 EU/mg.

PPD-B- or CE-induced IP-10 and IL-17A transcripts were significantly increased in *M. bovis*-infected cows relative to those in uninfected ones, and these levels were also higher than those of IL-6, IL-12, and TNF-α, and correlated well with the levels of IFN-γ. Therefore, the levels of PPD-B- and CE-induced IP-10 and IL-17A mRNA in PBMCs from animals experimentally infected with *M. bovis* 68002 were analyzed. The levels of PPD-B- and CE-induced IP-10 and IL-17A protein in plasma from animals experimentally infected with *M. bovis* 68002 were also analyzed.

### IP-10 and IL-17A Response to *M. bovis* Infection and Comparison with that of IFN-γ

**Figure 2:**
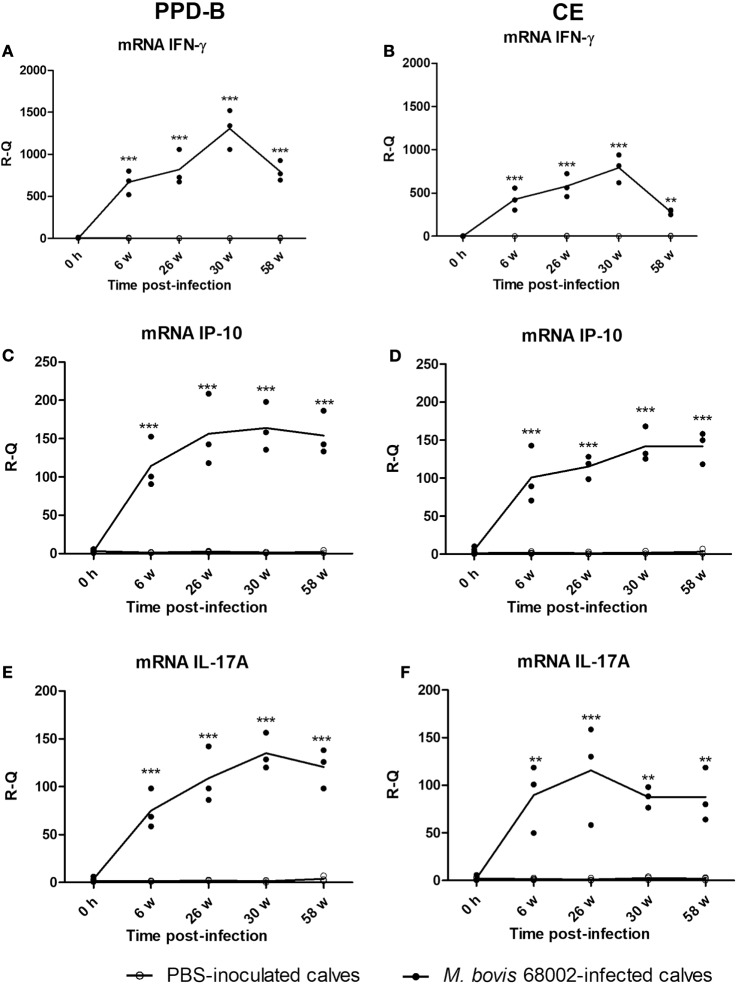
Levels of cytokines mRNA in *Mycobacterium bovis*-infected cattle. **(A)** Interferon gamma (IFN-γ) induced by PPD-B. **(B)** IFN-γ induced by CE. **(C)** IFN-γ-induced protein 10 (IP-10) induced by PPD-B. **(D)** IP-10 induced by CE. **(E)** IL-17A induced by PPD-B. **(F)** IL-17A induced by CE. Peripheral blood mononuclear cells (PBMCs) were isolated from calves experimentally infected with *M. bovis* (*n* = 3) and those inoculated with phosphate-buffered saline (PBS) (uninfected controls, *n* = 3) at 0 h and 6, 24, 30, and 58 weeks post-infection. PBMCs were then stimulated with PPD-B, CE, or PBS for 6 h. RNA was isolated to measure IFN-γ, IP-10, and IL-17A gene expression levels using the 2^−ΔΔ^*^CT^* method, with PBS-stimulated PBMCs used as a calibrator, and *β-actin* as the reference gene. The significance of differences between cytokine transcript levels in *M. bovis* 68002-infected calves and uninfected controls was determined at each time point using a two-way analysis of variance followed by Bonferroni post-test. **p < 0.001; ***p < 0.0001. Data are each replicate and means connected by lines (for *M. bovis*-infected cattle, *n* = 3; for uninfected cattle, *n* = 3).

**Figure 3:**
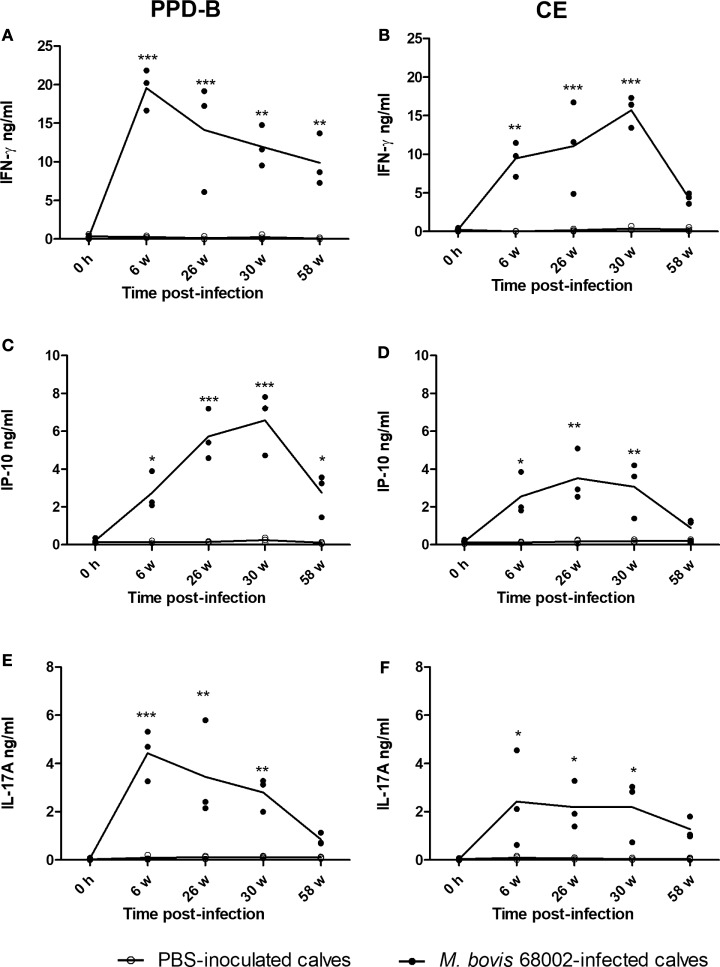
Level of cytokine proteins in *Mycobacterium bovis*-infected cattle. **(A)** Interferon gamma (IFN-γ) induced by PPD-B. **(B)** IFN-γ induced by CE. **(C)** IP-10 induced by PPD-B. **(D)** IP-10 induced by CE. **(E)** IL-17A induced by PPD-B. **(F)** IL-17A induced by CE. Whole blood was collected from three *M. bovis* 68002-infected and three phosphate-buffered saline (PBS)-inoculated calves, and then stimulated with PPD-B, CE, or PBS for 24 h, and at 0 h and 6, 26, 30, and 58 weeks post-infection. Plasma was harvested to measure IFN-γ, IP-10, and IL-17A expression levels using commercial kits. The significance of differences between cytokine expression levels in *M. bovis* 68002-infected calves and uninfected controls was determined at each time point using a two-way analysis of variance followed by Bonferroni post-test. *p < 0.05; **p < 0.001; ***p < 0.0001. Data are each replicate and means connected by lines (for *M. bovis*-infected cattle, *n* = 3; for uninfected cattle, *n* = 3).

To verify whether PPD-B- or CE-induced IP-10 and IL-17A expression was related to *M. bovis* infection, three Luxi beef calves were intravenously injected with *M. bovis* 68002, and three other calves were injected PBS as uninfected controls. The three *M. bovis* 68002-infected calves were confirmed to be *M. bovis* infected by TST, CFP-10/ESAT-6/TB10.4 protein cocktail-based skin test, IGRA and CE-based IGRA (Figures S1 and S2 in Supplementary Material), and the PBS-inoculated calves were confirmed to be uninfected with *M. bovis* (Figures S1 and S2 in Supplementary Material). The mRNA and protein expression levels of IFN-γ, IP-10, and IL-17A induced by PPD-B, CE, PET, or PBS were measured by real-time PCR and ELISA before injection and at 6, 26, 30, and 58 weeks post-infection. The mRNA and protein expression levels of IFN-γ, IP-10, and IL-17A induced by PET in PBMCs from both *M. bovis* 68002-infected and uninfected calves were close to those induced by PBS (data not shown). The levels of IFN-γ, IP-10, and IL-17A (mRNA and protein) induced by PET and PBS in *M. bovis* 68002-infected calves and uninfected calves were not significantly different (data not shown). After *M. bovis* 68002 infection, the responses of IP-10 and IL-17A (mRNA and protein) to PPD-B and CE showed kinetics similar to those of IFN-γ responses (Figures 2 and 3). The mRNA transcript levels of IFN-γ, IP-10, and IL-17A induced by PPD-B or CE in PBMCs from *M. bovis*-infected calves were significantly higher than those from uninfected calves between 6 and 58 weeks post-infection (Figure 2). However, the levels of IFN-γ (induced by CE) and IP-10 (induced by CE) protein at 58 weeks post-infection and IL-17A (induced by PPD-B or CE) protein at 58 weeks post-infection in *M. bovis*-infected calves showed no differences from those in uninfected calves (Figure 3). The IFN-γ (mRNA and protein) elicited by PPD-B and CE in *M. bovis*-infected calves were higher than those of IP-10 and IL-17A. The protein levels of IFN-γ ranged from 0 to 22 ng/ml, whereas IP-10 and IL-17A levels ranged from 0 to 7 ng/ml. Correlations between IFN-γ, IP-10, and IL-17A levels were determined at all treatments and all time points (*n* = 30 [6 cattle × 5 time points]). As shown in Table 4, IFN-γ, IP-10, and IL-17A levels showed good correlation with each other (*r* > 0.65). IFN-γ showed better correlation with IP-10 than with IL-17A. Therefore, PPD-B- and CE-induced IP-10 and IL-17A mRNA expression could be used to differentiate *M. bovis*-infected calves from uninfected ones between 6 and 58 weeks post-infection, whereas PPD-B- and CE-induced IP-10 and IL-17A protein expression could be used to differentiate *M. bovis*-infected calves from uninfected ones between 6 and 30 weeks post-infection.

**Table 4:** Correlations between interferon gamma (IFN-γ), IFN-γ-induced protein 10 (IP-10), and IL-17A (mRNA and proteins) induced by PPD-B and CE.

Correlation coefficient (Spearman *r*)	PPD-B-stimulated peripheral blood mononuclear cells (PBMCs) (mRNA)			CE-stimulated PBMCs (mRNA)			PPD-B-stimulated whole blood (protein)			CE-stimulated whole blood (protein)		
	
	IFN-γ	IP-10	IL-17A	IFN-γ	IP-10	IL-17A	IFN-γ	IP-10	IL-17A	IFN-γ	IP-10	IL-17A
IFN-γ		0.82	0.77		0.86	0.83		0.76	0.69		0.85	0.73
IP-10	0.82		0.73	0.86		0.87	0.76		0.72	0.85		0.66
IL-17A	0.77	0.73		0.83	0.87		0.69	0.72		0.73	0.66	

*PBMCs were isolated from three *Mycobacterium bovis* 68002-infected and three phosphate-buffered saline (PBS) inoculated calves, and then stimulated with PPD-B, CE, PET, or PBS for 6 h, at 0 h and 6, 26, 30, and 58 weeks post-infection. RNA was isolated to measure levels of IFN-γ, IP-10, and IL-17A gene expression using the 2^−ΔΔ^*^CT^* method, with PBS-stimulated PBMCs as a calibrator, and *β-actin* as the reference gene. Whole blood was collected from three *M. bovis* 68002-infected and three PBS-inoculated calves, and then stimulated with PPD-B, CE, PET, or PBS for 24 h, at 0 h and 6, 26, 30, and 58 weeks post-infection. Plasma was harvested to measure levels of IFN-γ, IP-10, and IL-17A expression using commercial kits. Correlation coefficients were determined using Spearman’s two-tailed correlation test. All correlations were significant (*p* < 0.05). The comparisons included all treatments and all time points (*n* = 30 [6 cattle × 5 time points]). PPD-B: Bovine Tuberculin PPD, 300 µg/ml (Prionics AG, Schlieren, Switzerland). CE, CFP-10-ESAT-6, 20 μg/ml, expressed and purified in our lab, with a Trx-His-S tag at the N-terminus, with endotoxin at a concentration less than 10 EU/mg*.

To confirm infection with *M. bovis* 68002, three *M. bovis* 68002-infected calves were slaughtered after experiment. Only one calve showed typically tubercles on the lungs, and there were no typical tubercles on the livers, spleen or other organs. So, we collected three pieces of each tissue (lung, liver, kidney, spleen, and bronchial lymph nodes) for nested PCR, *Mycobacterium* culture, and hematoxylin–eosin staining. The lung and liver tissues were positive by nested PCR and *Mycobacterium* culture, hematoxylin–eosin staining showed no pathological changes in the livers, kidneys, or spleens.

### Comparisons of Cytokines between Nested PCR-P Cattle and PCR-N Cattle

**Figure 4:**
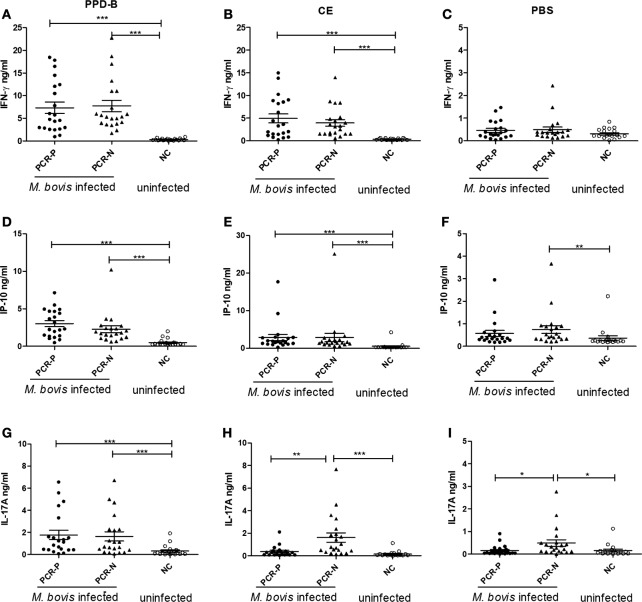
The levels of cytokine protein expression in PCR-P, PCR-negative (PCR-N), and NC. **(A)** Interferon gamma (IFN-γ) induced by PPD-B. **(B)** IFN-γ induced by CE. **(C)** IFN-γ induced by phosphate-buffered saline (PBS). **(D)** IFN-γ-induced protein 10 (IP-10) induced by PPD-B. **(E)** IP-10 induced by CE. **(F)** IP-10 induced by PBS. **(G)** IL-17A induced by PPD-B. **(H)** IL-17A induced by CE. **(I)** IL-17A induced by PBS. PCR-P (nested PCR positive): 21 cattle from a dairy where bovine tuberculosis (bTB) was prevalent and *Mycobacterium bovis* infection was detected by tuberculin skin test (TST), CFP-10/ESAT-6/TB10.4 protein cocktail-based skin test, IGRA, CE-based IGRA, and nested PCR. PCR-N (nested PCR-N): 21 cattle from a dairy where bTB was prevalent and *M. bovis* infection was detected by TST, CFP-10/ESAT-6/TB10.4 protein cocktail-based skin test, IGRA, and CE-based IGRA, but not detected by nested PCR. NC (uninfected control): 20 cattle from a bTB-free diary determined *M. bovis* negative by TST, CFP-10/ESAT-6/TB10.4 protein cocktail-based skin test, IGRA, CE-based IGRA, and nested PCR. Whole blood was collected and stimulated with PPD-B, CE, or PBS for 24 h. Plasma from each well was harvested to measure levels of IFN-γ, IP-10, and IL-17A expression using commercial kits. The significance of differences between cytokine expression levels in PCR-P, PCR-N, and NC was determined using a one-way analysis of variance (Kruskal–Wallis test) followed by Dunn’s multiple comparison. *p < 0.05; **p < 0.001; ***p < 0.0001.

To assess whether IP-10 and IL-17A proteins could be used to detect all naturally *M. bovis*-infected cattle, more than 1,000 cows were detected by TST, CFP-10/ESAT-6/TB10.4 protein cocktail-based skin test, IGRA, and CE-based IGRA. 151 *M. bovis*-infected cows from dairies where bTB was prevalent and 50 uninfected cows from bTB-free dairies were selected for follow-up study. Nasal swabs from these 201 selected cows were collected and tested by nested PCR. 35 of 151 cows were determined positive by nested PCR, and 21 of these cows were confirmed positive by *Mycobacteria* culture and classified as PCR-P. The remaining 116 of 151 cows were determined negative by nested PCR and classified as PCR-N (data not shown). 21 PCR-P cows, 21 PCR-N cows, and 20 uninfected cows (NC) were randomly selected for additional analysis. Heparinized whole blood was collected from each cow and then stimulated by PPD-B, PPD-A, CE, or PBS for 24 h. The levels of IFN-γ, IP-10, and IL-17A protein in stimulated plasma were determined by commercial kits (Figure 4). PPD-B-induced IFN-γ, and IL-17A levels in the PCR-P and PCR-N groups were higher than those induced by CE, and significantly higher than those in the NC group. IFN-γ and IP-10 levels induced by CE in the PCR-P and PCR-N groups were significantly higher than those in the NC group. PBS-induced IP-10 levels were significantly higher in the PCR-N group than in the PCR-P and NC groups. PBS-induced IFN-γ levels were similar in the PCR-P, PCR-N, and NC groups. However, CE- and PBS-induced IL-17A levels were significantly higher in the PCR-N group than in the PCR-P and NC groups. Correlations among IFN-γ, IP-10, and IL-17A protein levels were calculated. This comparison included all individuals and all treatments (*n* = 62 [21 PCR-P cows + 21 PCR-N cows + 20 NC cows]). As shown in Table 5, IFN-γ, IP-10, and IL-17A induced by PPD-B or CE were significantly correlated with one other. PBS-induced IFN-γ levels showed no correlation with those of PBS-induced IP-10 (*r* = 0.12, *p* > 0.05) and IL-17A (*r* = −0.10, *p* > 0.05), whereas PBS-induced IP-10 showed a weak correlation with IL-17A (*r* = 0.38, *p* < 0.05).

**Table 5:** Correlations between interferon gamma (IFN-γ), IFN-γ-induced protein 10 (IP-10), and IL-17A (proteins) induced by PPD-B and CE.

Correlation coefficient (Spearman *r*)	PPD-B-stimulated whole blood (protein)			CE-stimulated whole blood (protein)			Phosphate-buffered saline (PBS)-stimulated whole blood (protein)		
	IFN-γ	IP-10	IL-17A	IFN-γ	IP-10	IL-17A	IFN-γ	IP-10	IL-17A
IFN-γ		0.59*	0.62*		0.65*	0.52*		0.12	0.38*
IP-10	0.59*		0.54*	0.65*		0.45*	0.12		−0.10
IL-17A	0.62*	0.54*		0.52*	0.45*		0.38*	−0.10	

Whole blood was isolated from 21 PCR-P (nested PCR positive), 21 PCR-N (nested PCR negative) and 20 NC (uninfected control) cattle and then stimulated with PPD-B, CE, or PBS for 24 h. Plasma was harvested to measure levels of IFN-γ, IP-10, and IL-17A protein expression using commercial kits. Correlation coefficients were determined using Spearman’s two-tailed correlation test. *p < 0.05, significant correlation. Comparisons included all individuals and all treatments (n = 62 [21 PCR-P + 21 PCR-N + 20 NC]). PCR-P: cattle from a dairy where bTB was prevalent, and M. bovis infection was detected by TST, CFP-10/ESAT-6/TB10.4 protein cocktail-based skin test, IGRA, CE-based IGRA, and nested PCR. PCR-N: cattle from a dairy where bTB was prevalent, and M. bovis infection was detected by TST, CFP-10/ESAT-6/TB10.4 protein cocktail-based skin test, IGRA and CE-based IGRA, but undetected by nested PCR. NC: cattle from a bTB-free diary and determined M. bovis negative by TST, CFP-10/ESAT-6/TB10.4 protein cocktail-based skin test, IGRA, CE-based IGRA, and nested PCR. PPD-B: Bovine Tuberculin PPD, 300 μg/ml (Prionics AG, Schlieren, Switzerland). CE: CFP-10-ESAT-6, 20 μg/ml, expressed and purified in our lab, with a Trx- His-S tag at the N-terminus, with endotoxin at a concentration less than 10 EU/mg.

To confirm infection, one naturally *M. bovis*-infected cow that was found positive by TST, CFP-10/ESAT-6/TB10.4 protein cocktail-based skin test, IGRA, CE-based IGRA, and nested PCR, was slaughtered after the experiment. There were no typical tubercles on the lung, kidney, and spleen, and hematoxylin–eosin staining of lung, kidney, spleen, and bronchial lymph node tissues indicated no pathological changes. However, the nasal swab and BAL fluid were positive by nested PCR.

## Discussion

### CE-Induced IL-17A Was Not a Reliable Indicator of Mycobacteria Shedding in Cattle

The airborne route of infection is considered as the most common for the transmission of *M. bovis*, and more than 15% of cattle with bTB shed the mycobacteria, mainly at an early stage in infection. Nested PCR based on the amplification of *mpb*70 can be used to detect *Mycobacteria* in milk, nasal exudates, and BAL fluid. Previous studies using nested PCR showed that 26% of TST-reactors shed *M. bovis* in nasal exudates (12). Furthermore, Flores-Villalva et al. found that in a high-prevalence herd, 60.3% (38/63) of TST-positive and 63.4% (40/63) of ESAT-6-CFP-10-based skin test-positive cattle were confirmed positive for *M. bovis* infection by nested PCR, whereas in a low-prevalence herd, 87.5% (7/8) of the ESAT-6-CFP-10-based skin test-positive cattle were confirmed positive by nested PCR (11). However, 24.84% (205/825) of *M. bovis-*infected cattle were positive by nested PCR in our previous study (9), and we found 23.18% (35/151) of *M. bovis-*infected cattle were positive by nested PCR in current study. The lower positivity rate in our study may be related to a difference in the bTB prevalence at diaries or the greater number of animals included in this study. There are few data on the differences in immune responses or immunopathology between cattle determined PCR positive and negative for *M. bovis* infection (*M. bovis*-infected animals diagnosed by TST or IGRA), but we did find the CE-induced and uninduced IL-17A (protein) levels were significantly higher in PCR-N cattle than in PCR-P and uninfected cattle (Figure 4). It indicated that *M. bovis*-infected cattle could be divided into two stages by using TST or IGRA and nested PCR results. One cow naturally infected with *M. bovis* in the PCR-P group was slaughtered and showed a low level of CE-induced IL-17A in plasma and no lesions in the lungs. The reason for this phenomenon is not known, but recent studies have shown that IL-17A (mRNA and protein) may be predictive of both vaccine efficacy and lesion severity when measured after vaccination and during infection, respectively (13, 14). Blanco et al. also found that increased PPD-B induced IL-17A transcripts in PBMC is associated with pathology in *M. bovis*-infected cattle: cattle with macroscopic lesions showed a higher level of IL-17A transcripts than animals without macroscopic lesions (13). Therefore, we speculate that *M. bovis*-infected cattle that were PCR positive and those that were negative may be at different stages in the progression of bTB and might show different immune responses to *M. bovis-*specific antigens. More studies are needed to test this hypothesis.

Our study also showed that the levels of PPD-B-stimulated IL-17A (mRNA and protein) were significantly higher in cattle naturally or experimentally infected with *M. bovis* than in those that were uninfected. The levels of PPD-B-induced IL-17A (mRNA and protein) could be used to differentiate *M. bovis*-infected calves from uninfected ones for 6 to 30 weeks post-infection. However, CE-induced IL-17A (protein) was not a reliable indicator of *M. bovis* infection in cattle that were confirmed positive for infection by nested PCR, and levels of IL-17A (mRNA and protein) were lower than those of IFN-γ. Together, these findings suggest that IL-17A is not suitable for the diagnosis of bTB.

### Limitations of IP-10 As Biomarker of bTB

Several recent studies have shown that mycobacterial antigen (PPD-B, CFP-10-ESAT-6) -stimulated IP-10 levels are higher in patients with active TB and latent TB compared with healthy controls and to IFN-γ (15, 16). Unlike IFN-γ, IP-10 levels were not age dependent, and more TB cases were identified in children aged <5 years when this chemokine was used for detection (17–19). This chemokine can even be used to detect TB in patients with HIV and those undergoing tuberculosis therapy (20–22). Therefore, IP-10 can be used as a biomarker for TB. Similarly, Goosen et al. found that in buffaloes naturally infected with *M. bovis*, the levels of IP-10 protein expression were significantly elevated in whole blood stimulated with ESAT-6/CFP-10 and higher than those of IFN-γ, suggesting its potential as a biomarker for bTB in African buffaloes (7, 23). Waters et al. found that PPD-B specific IP-10 mRNA showed a pattern of expression similar to that IFN-γ mRNA over the entire course of *M. bovis* infection and could be a biomarker for bTB, and IP-10 protein showed a poor correlation with IFN-γ in cattle experimentally infected with *M. bovis* (24). By contrast, Parsons et al. found that levels of mycobacterial-specific IP-10 protein strongly correlated with those of IFN-γ in cattle naturally infected with *M. bovis*, and that the differential release of IP-10 induced by PPD-B and PPD-A could be used to distinguish between *M. bovis-*infected cattle and bTB-free cattle with a sensitivity of 100% (95% Cl, 86–100%) and specificity of 97% (95% Cl, 85–100%) (25). In our study, the levels of IP-10 mRNA and protein induced by PPD-B or CE were significantly higher in cattle naturally or experimentally infected with *M. bovis* compared with those that were bTB free, which is consist with Parsons’s results but contrasts with the results of Waters. This may because a bovine IP-10 ELISA kit was used in our study and the study by Parsons et al. to determine IP-10 levels, whereas a human IP-10 ELISA kit was used in the Waters et al. study.

Previous studies have shown that levels of IP-10 induced by PPD-B or CE were significantly higher than those in IFN-γ in patients with TB and in *M. tuberculosis*-infected monkeys (26), whereas, in our study, levels of antigen-induced IP-10 were lower than those of IFN-γ in *M. bovis*-infected cattle. Because the amount of IP-10 in *M. bovis-*infected cattle and healthy cattle is rarely reported, the differences in results between humans, monkeys, and cattle may be related to differences in the species tested or the limited number of animals included in our study. The lower levels of PPD-B- or CE-induced IP-10 protein relative to that IFN-γ limit the utility of IP-10 for the diagnosis of bTB. The levels of PPD-B- or CE-induced IP-10 transcripts, however, should be analyzed further for their value in bTB diagnosis.

We also found that levels of PPD-B- or CE-stimulated IP-10 in PCR-P animals were similar to those in PCR-N animals, and we could not differentiate PCR-P from PCR-N animals with this chemokine. However, levels of unstimulated IP-10 (PBS exposed) in PCR-N animals were significantly higher than those in uninfected animals but similar to PCR-P animals. Similarly, studies on human TB have shown that antigen-stimulated IP-10 in plasma cannot be used to distinguish between patients with active and latent TB, whereas serum IP-10 concentrations were higher in patients with active TB than in those with latent TB. However, IP-10 is likely to be a non-specific marker of inflammation and also elevated in other bacterial, viral, and parasitic infections, whereas unstimulated serum IP-10 levels primarily reflect a pro-inflammatory state, therefore, unstimulated IP-10 may be limited in its use for detection of PCR-N cattle.

One drawback of this study is that we did not test every cow naturally or experimentally infected with *M. bovis* by nested PCR. Thus, we could not evaluate the mRNA transcript levels of cytokines in PCR-P and PCR-N animals. Another limitation is that it was impossible to slaughter all cattle used in this study to confirm the stages of bTB progression in the *M. bovis*-infected cattle (either PCR positive or negative). Therefore, more work is needed to determine the differences in immune responses and immunopathology between PCR-P and PCR-N cattle.

## Conclusion

CE-induced IL-17A protein cannot detect all *M. bovis*-infected cattle, and PPD-B- or CE-induced IP-10 and IL-17A protein in *M. bovis*-infected cattle were lower than IFN-γ protein. IL-17A and IP-10 proteins are not suitable as biomarkers for bovine tuberculosis, but the levels of PPD-B- or CE-induced IP-10 mRNA transcripts should be analyzed further for their potential to be used in the diagnosis of bTB.

## Author Contributions

HZ, TX, JD and HJ designed the experiments. TX, XG, HY, PL, QL, XS, SH, XG and WY performed the experiments and analyzed the data. TX and XG wrote and revised the paper.

## Conflict of Interest Statement

The authors declare that the research was conducted in the absence of any commercial or financial relationships that could be construed as a potential conflict of interest.

## Supplementary Material

Figure S1 *Mycobacterium bovis* 68002 infection confirmed by skin test. **(A)** *M. bovis* 68002 infection confirmed by tuberculin skin test (TST). **(B)** *M. bovis* 68002 infection confirmed by CFP-10/ESAT-6/TB10.4 protein cocktail-based skin test. Three *M. bovis* 68002-infected calves and three PBS-inoculated calves were tested using TST and CFP-10/ESAT-6/TB10.4 protein cocktail-based skin test at 8 and 32 weeks post-infection. The TST was performed as the Chinese diagnostic standard for bovine tuberculosis (GB/T 18645-2002), and the CFP-10/ESAT-6/TB10.4 protein cocktail-based skin test was previously established in our laboratory. PPD-B (PPD-B, 2,500 IU/cattle) and the CFP-10/ESAT-6/TB10.4 protein cocktail were intradermally injected (0.1 ml each) into two sites on the same side of a cow’s neck. Differences in skin thicknesses (mm) pre- and 72 h post-injection were calculated. With the GB/T 18645-2002, if the difference in skin thicknesses was ≥4 mm, the cattle were considered as *M. bovis*-infected; if the difference in skin thicknesses was <2 mm, the cattle were considered *M. bovis*-uninfected. For the CFP-10/ESAT-6/TB10.4 protein cocktail-based skin test, if the difference in skin thicknesses was ≥1.1 mm, the cattle were considered *M. bovis* infected; if the difference in skin thicknesses was <1.1 mm, the cattle were considered free from bTB. PPD-B: Bovine tuberculin (Harbin Pharmaceutical Group, Heilongjiang Province, China), 2,500 IU/cattle. CFP-10/ESAT-6/TB10.4 protein cocktail: 0.5 mg/ml, endotoxin less than 10 EU/mg, prepared in the Institute of Animal Sciences (IAS-CAAS).

Figure S2 *Mycobacterium bovis* 68002 infection confirmed by IGRA. **(A)** *M. bovis* 68002 infection confirmed by IGRA [interferon gamma (IFN-γ) release assay], differences in OD value PPD-B- and PPD-A-stimulated blood plasma. **(B)** *M. bovis* 68002 infection confirmed by IGRA, differences in OD value PPD-B- and phosphate-buffered saline (PBS)-stimulated blood plasma. **(C)** *M. bovis* 68002 infection confirmed by CE-based IGRA (CFP-10/ESAT-6-based IFN-γ release assay), differences in OD value CE- and PBS-stimulated blood plasma. Three *M. bovis* 68002-infected calves and three PBS-inoculated calves were tested using IGRA and CE-based IGRA before injection, and at 8, 24, 48, 72, and 96 h, and 2, 4, 6, 8, 12, 16, 20, 26, 30, 34, 38, 45, 50, and 58 weeks post-infection. For IGRA, if PPD-B-simulated blood plasma having an OD value more than 0.100 above that of plasma stimulate with PPD-A and PBS indicated cattle were *M. bovis* infected. For CE-based IGRA, if CE-stimulated blood plasma having an OD value more than 0.100 above that of plasma stimulated with PBS indicated cattle were *M. bovis*-infected. PPD-B: Bovine Tuberculin PPD, 300 µg/ml (Prionics AG, Schlieren, Switzerland). CE: CFP-10-ESAT-6, 20 μg/ml, expressed and purified in our lab, with a Trx-His-S tag at the N-terminus, with endotoxin at a concentration less than 10 EU/mg.
