# Supplementary figures and images for: Limitations of Using IL-17A and IFN-γ-Induced Protein 10 to Detect Bovine Tuberculosis
Source: Front Vet Sci. 2018 Mar 6;5:28. doi: 10.3389/fvets.2018.00028 (PMC5845669; doi:10.3389/fvets.2018.00028)

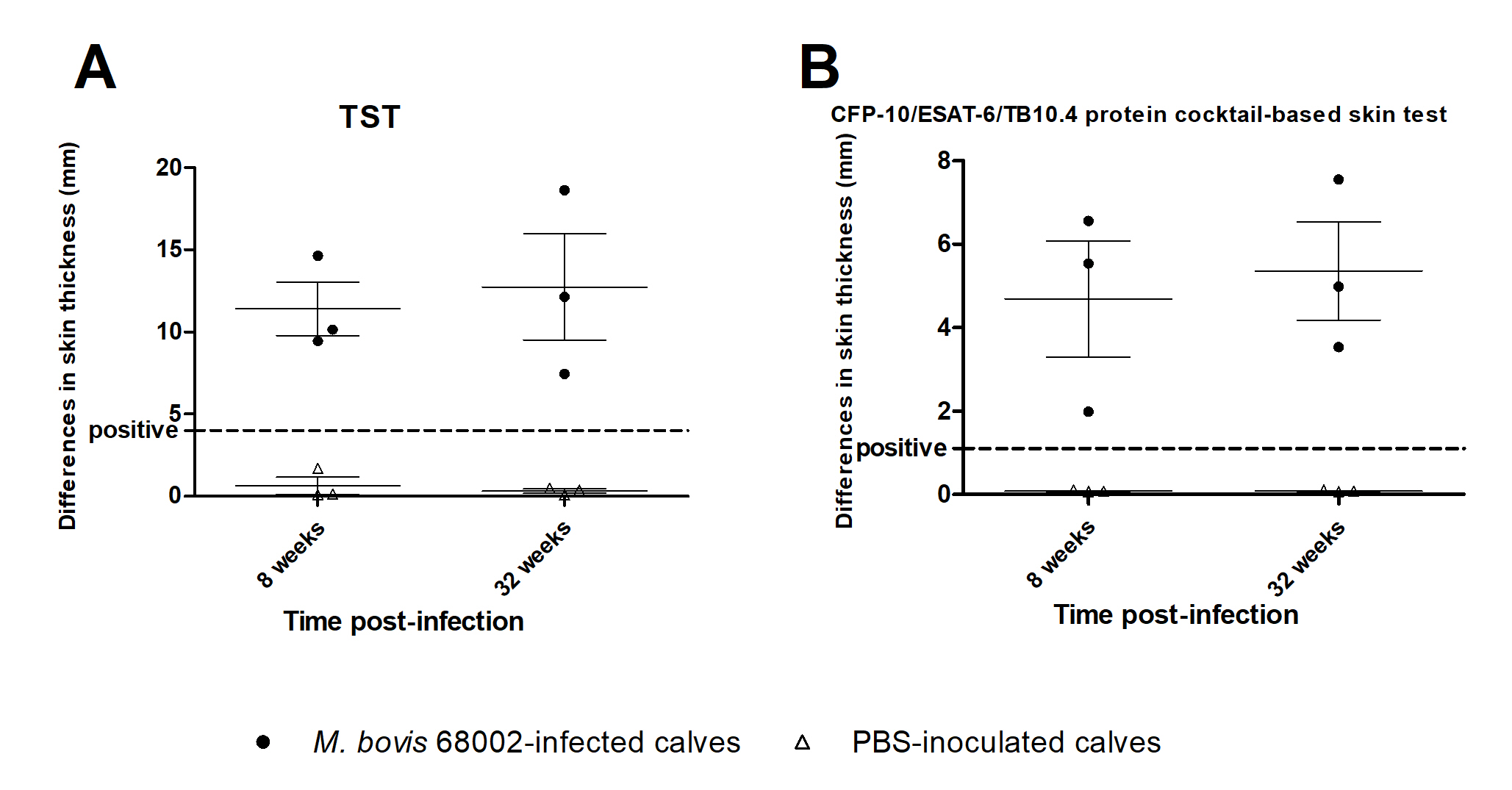

Supplement: Figure S1 — Mycobacterium bovis 68002 infection confirmed by skin test. (A) M. bovis 68002 infection confirmed by tuberculin skin test (TST). (B) M. bovis 68002 infection confirmed by CFP-10/ESAT-6/TB10.4 protein cocktail-based skin test. Three M. bovis 68002-infected calves and three PBS-inoculated calves were tested using TST and CFP-10/ESAT-6/TB10.4 protein cocktail-based skin test at 8 and 32 weeks post-infection. The TST was performed as the Chinese diagnostic standard for bovine tuberculosis (GB/T 18645-2002), and the CFP-10/ESAT-6/TB10.4 protein cocktail-based skin test was previously established in our laboratory. PPD-B (PPD-B, 2,500 IU/cattle) and the CFP-10/ESAT-6/TB10.4 protein cocktail were intradermally injected (0.1 ml each) into two sites on the same side of a cow’s neck. Differences in skin thicknesses (mm) pre- and 72 h post-injection were calculated. With the GB/T 18645-2002, if the difference in skin thicknesses was ≥4 mm, the cattle were considered as M. bovis-infected; if the difference in skin thicknesses was <2 mm, the cattle were considered M. bovis-uninfected. For the CFP-10/ESAT-6/TB10.4 protein cocktail-based skin test, if the difference in skin thicknesses was ≥1.1 mm, the cattle were considered M. bovis infected; if the difference in skin thicknesses was <1.1 mm, the cattle were considered free from bTB. PPD-B: Bovine tuberculin (Harbin Pharmaceutical Group, Heilongjiang Province, China), 2,500 IU/cattle. CFP-10/ESAT-6/TB10.4 protein cocktail: 0.5 mg/ml, endotoxin less than 10 EU/mg, prepared in the Institute of Animal Sciences (IAS-CAAS). [file image_1.JPEG]

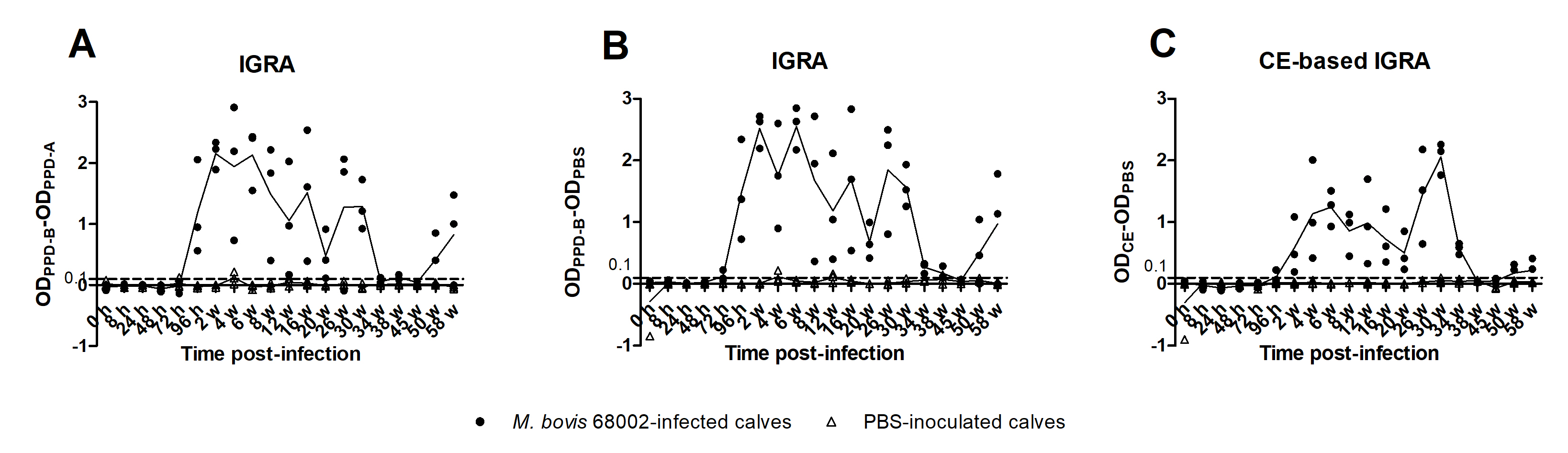

Supplement: Figure S2 — Mycobacterium bovis 68002 infection confirmed by IGRA. (A) M. bovis 68002 infection confirmed by IGRA [interferon gamma (IFN-γ) release assay], differences in OD value PPD-B- and PPD-A-stimulated blood plasma. (B) M. bovis 68002 infection confirmed by IGRA, differences in OD value PPD-B- and phosphate-buffered saline (PBS)-stimulated blood plasma. (C) M. bovis 68002 infection confirmed by CE-based IGRA (CFP-10/ESAT-6-based IFN-γ release assay), differences in OD value CE- and PBS-stimulated blood plasma. Three M. bovis 68002-infected calves and three PBS-inoculated calves were tested using IGRA and CE-based IGRA before injection, and at 8, 24, 48, 72, and 96 h, and 2, 4, 6, 8, 12, 16, 20, 26, 30, 34, 38, 45, 50, and 58 weeks post-infection. For IGRA, if PPD-B-simulated blood plasma having an OD value more than 0.100 above that of plasma stimulate with PPD-A and PBS indicated cattle were M. bovis infected. For CE-based IGRA, if CE-stimulated blood plasma having an OD value more than 0.100 above that of plasma stimulated with PBS indicated cattle were M. bovis-infected. PPD-B: Bovine Tuberculin PPD, 300 µg/ml (Prionics AG, Schlieren, Switzerland). CE: CFP-10-ESAT-6, 20 μg/ml, expressed and purified in our lab, with a Trx-His-S tag at the N-terminus, with endotoxin at a concentration less than 10 EU/mg. [file image_2.JPEG]
